# Integrative Analysis of Placental Methylomes Identifies Epigenetically Regulated Genes Implicated in Fetal Growth Restriction

**DOI:** 10.3390/ijms27031448

**Published:** 2026-01-31

**Authors:** Magdalena Bednarek-Jędrzejek, Olga Taryma-Leśniak, Małgorzata Poniatowska, Mateusz Cejko, Katarzyna Maksym, Sylwia Dzidek, Małgorzata Blatkiewicz, Ewa Kwiatkowska, Andrzej Torbé, Sebastian Kwiatkowski

**Affiliations:** 1 Department of Gynecology and Obstetrics, Pomeranian Medical University, 70-111 Szczecin, Poland; sylwiadzidek@wp.pl (S.D.); torbea@wp.pl (A.T.); kwiatkowskiseba@gmail.com (S.K.); 2 Department of Biochemical Research, Pomeranian Medical University, 71-460 Szczecin, Poland; olga.taryma.lesniak@pum.edu.pl; 3 Department of Nuclear Medicine, Pomeranian Medical University, 71-252 Szczecin, Poland; m.poniatowska94@gmail.com; 4 Pomeranian Medical University, 70-204 Szczecin, Poland; 42348@student.pum.edu.pl; 5 UCL Elizabeth Garrett Anderson Institute for Women’s Health, University College London, London WC1E 6HU, UK; k.maksym@ucl.ac.uk; 6 Department of Histology and Embryology, Poznan University of Medical Sciences, 60-781 Poznan, Poland; mblatkiewicz@ump.edu.pl; 7 Department of Nephrology, Transplantology and Internal Medicine, Pomeranian Medical University, 70-111 Szczecin, Poland; ewakwiat@gmail.com

**Keywords:** fetal growth restriction, FGR, DNA methylation, epigenetics, epigenome

## Abstract

Fetal growth restriction (FGR) is a major contributor to perinatal morbidity and mortality, most commonly arising from placental dysfunction, with increasing evidence implicating aberrant DNA methylation in its pathogenesis. To identify robust epigenetic alterations associated with FGR, we analyzed placental chorionic villi from an in-house early-onset FGR cohort and compared them with a publicly available dataset (GSE100197). DNA methylation profiling was performed using Illumina EPIC (in-house) and 450K (public) arrays, processed with identical normalization and quality-control pipelines, including adjustment for gestational age and estimation of placental cell-type composition. Differentially methylated positions (DMPs) were identified using linear regression models, revealing 10,427 DMPs in the in-house cohort and 7467 in the public dataset, with 108 shared DMPs showing consistent direction of change across both cohorts. Promoter-associated DMPs were mapped to genes involved in angiogenesis, morphogenesis, immune regulation, and transcriptional control, including *EPHA1*, *ANGPTL6*, *ITGAX*, *BCL11B*, and *CYP19A1*, while additional novel candidates such as *SLC39A12*, *YEATS4*, and *MIR515* family members were also identified. Functional annotation suggests that these methylation changes may influence pathways essential for placental vascular development and structural organization. Overall, this cross-cohort comparison highlights reproducible epigenetic signatures of FGR and underscores the need for standardized approaches to clarify the molecular mechanisms underlying placental insufficiency.

## 1. Introduction

Fetal growth restriction (FGR) is a condition in which the fetus fails to achieve the expected biological growth potential, as a result of factors that limit growth during pregnancy [1]. FGR affects around 5–10% of pregnancies, and is one of the leading causes of perinatal morbidity and mortality [2,3]. This condition is strongly associated with a higher risk of preterm delivery [4], which further compounds adverse outcomes due to the challenges of early gestational age (GA). Preterm infants with FGR are at increased risk of complications such as respiratory distress syndrome, necrotizing enterocolitis, and intraventricular hemorrhage [5], as well as long-term consequences including an elevated likelihood of developing metabolic syndrome, cardiovascular diseases, and neurodevelopmental impairments [6]. Furthermore, FGR significantly increases the costs of perinatal care, with expenses associated with the care of prematurely born infants with FGR constituting nearly 70% of the total expenses related to the treatment of all premature newborns [7,8,9,10].

Fetal growth disorders occur when one or more components of the maternal-fetal unit, which complement each other during physiological pregnancy, are disturbed. The disruption can originate from any of the three compartments, i.e., maternal, placental, or fetal. Among fetuses without genetic abnormalities, placental factors are the most frequent cause of FGR, accounting for approximately 30% of cases [3]. Placental development and function are influenced by numerous regulatory factors, with growing evidence highlighting the pivotal role of epigenetics in abnormal fetal growth [11]. Consequently, an increasing number of studies focus on the epigenetic changes implicated in the development of this disorder [12,13,14]. Epigenetic mechanisms play a critical role in the regulation of gene expression, which is essential for normal fetal development [15]. A variety of epigenetic factors, including DNA methylation, histone modifications, and chromatin-associated proteins, together with small RNA-mediated regulatory pathways, are known to interact with environmental fluctuations and regulate gene expression programs across developmental and physiological contexts [16].

One of the most investigated epigenetic mechanisms is DNA methylation, which is a covalent addition of a methyl group to cytosines in the genome [17]. Several studies describe the involvement of methylation changes in pregnancies complicated by FGR [18,19,20,21,22,23,24]. Nevertheless, the findings remain inconsistent, with each study identifying different methylation changes. This variability underscores the involvement of epigenetic changes in the development of FGR but highlights the need for standardization in experimental approaches, including patient selection and methodologies [25].

Although several studies have examined whole-genome DNA methylation changes in FGR complicated pregnancies [14,26,27,28], their interpretability and reproducibility are limited by heterogeneity in study design, analytical pipelines, and data availability across studies. In particular, only one publicly available dataset (GSE100197) provides raw IDAT files together with the study design [24] that enables direct cross-cohort comparison. In this study, we used a uniform experimental approach and data analysis to investigate CpG sites, whose methylation level was significantly altered in pregnancies complicated by FGR across independent cohorts. We identified a subset of CpG sites, consistently differentially methylated between cases and controls and localized to promoter regions of several well-established FGR-related genes involved in angiogenesis and placental development, such as *MMP9*, *EPHA1*, *ITGAX*, *ANGPTL6*, and *CYP19A1*. Among these, *CYP19A1* is the only gene for which methylation has previously been implicated in FGR [29], and our findings confirm its epigenetic involvement across independent datasets. In addition, we identified a set of previously unreported candidates to which methylation has not been linked to FGR to date, including *SLC39A12*, *BCL11B*, *YEATS4*, and *SEMA5A*.

Our findings not only confirm that methylation changes play a role in FGR development but also emphasize the critical need for standardizing studies to better investigate the molecular pathology of FGR.

## 2. Results

### 2.1. Placenta Samples in Both Study Groups Display Similar Cell Types Composition

**Figure 1:**
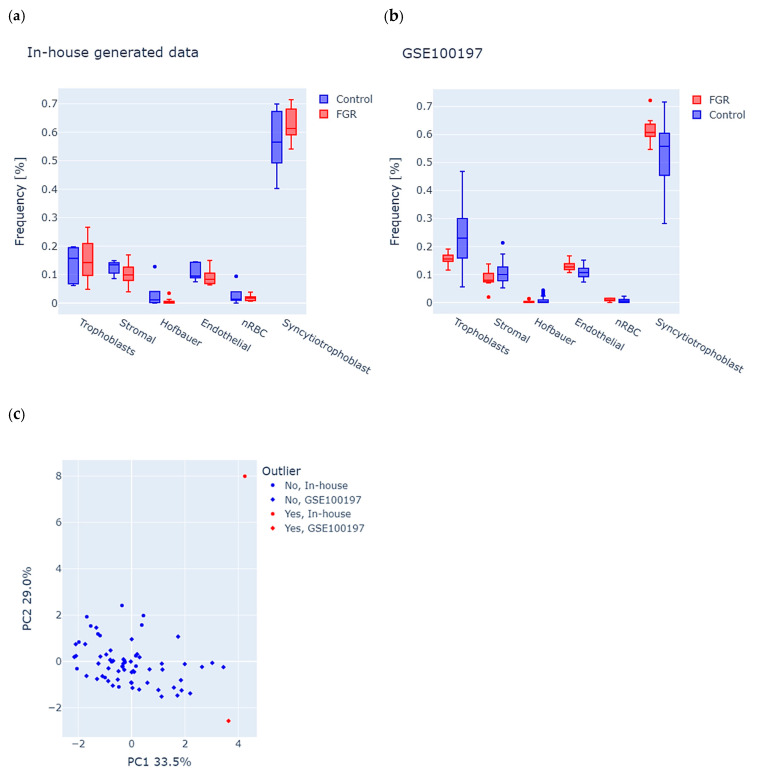
Analysis of tissue composition in placenta samples from fetal growth restriction (FGR) and controls in both the in-house and GSE100197 groups. (**a**,**b**) Predicted frequencies of six major placental cell types, including trophoblasts, stromal cells, Hofbauer cells, endothelial cells, nucleated red blood cells (nRBC), and syncytiotrophoblasts in (**a**) in-house and (**b**) GSE100197 samples. (**c**) Identification of outliers, according to abnormal cell-type composition. Each dot represents a sample, colored and shaped according to its origin (in-house or GSE100197) and outlier status. The dots highlighted in red indicate an outlier, suggesting potential bias of tissue composition on methylation analysis.

Considering the cell-type-specific methylation signatures in the placenta, we first used the RPC algorithm to analyze potential differences in tissue composition (see the Section 4 for details) between FGR and controls in each study group. We observed a similar distribution of cells in both datasets, with the largest percentage of syncytiotrophoblast cells, which is consistent with the normal distribution of cells in the placenta (Figure 1a,b). We further used estimated cell proportions and performed principal component analysis (PCA) of the combined in-house and GSE100197 groups to detect the samples with abnormal tissue composition (see the Section 4 for details). The first two principal components accounted for 33.5% (PC1) and 29.0% (PC2) of the total variance (Figure 1c), and enabled the identification of two outliers, one in-house control and one GSE100197 control, which were excluded from the study. Consequently, the final in-house dataset included 11 FGR (F = 7, M = 4) and 4 controls (F = 3, M = 1), and the GSE100197 dataset had 10 FGR (F = 6, M = 4) and 41 controls (F = 17, M = 24).

### 2.2. There Is a Subset of Differentially Methylated Positions Common Between Both Study Groups

In order to identify differences in DNA methylation between the cases and controls, we first focused on identifying potential confounding factors that could affect the results of the analysis. We did not recognize any confounders in the GSE100197 group. However, in contrast to the GSE100197 group, we identified gestational age as a considerable cofounder of the in-house group (Supplementary Figure S1). Specifically, GA was significantly different in this group between cases and controls (*p*-value ≤ 0.05, Kruskal–Wallis test).

**Figure 2:**
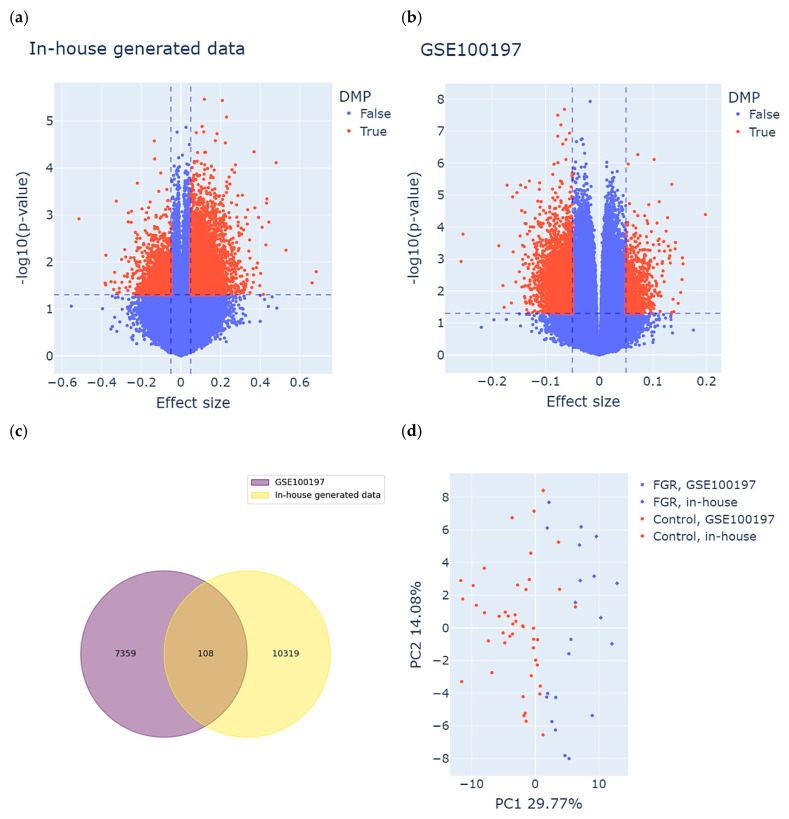
Identification of meaningful differentially methylated positions (DMPs) in both study groups. (**a**,**b**) Volcano plots displaying DMPs identified in the (**a**) in-house group and the (**b**) GSE100197 group. Each dot represents an individual CpG site. The X-axis shows the effect size (difference in methylation between FGR and control), and the Y-axis indicates the statistical significance, expressed as –log10(*p*-value). CpG sites meeting the DMP criteria are highlighted in red and were defined based on both statistical significance (*p*-value threshold, horizontal dashed line) and biological relevance (effect size threshold, vertical dashed lines). (**c**) DMPs common between the study groups, meeting both the definition of DMPs as well as consistency of methylation change in FGR compared to controls. (**d**) Clustering of the samples based on the identified subset of common DMPs. Each dot represents a sample, colored and shaped according to its origin (in-house or GSE100197) and clinical status (FGR or control).

Consequently, we used univariate linear regression models for the GSE100197 group and multivariate (adjusted for GA) for the in-house group to perform an epigenome-wide association analysis. Based on the definition of DMPs, we identified 10427 DMPs (8696 hyper- and 1731 hypomethylated) for the in-house group and 7467 DMPs (1295 hyper- and 6172 hypomethylated) for the GSE100197 group. The results are visualized in the volcano plot in Figure 2a,b. A total of 108 DMPs were common between both datasets and displayed the same direction of methylation change in both study groups (Figure 2c and Supplementary Table S1). Among these DMPs, 50 were hyper- and 58 hypomethylated in cases compared to controls in both study groups.

To assess the discriminatory power of the identified subset of DMPs, we performed PCA on both the FGR and control samples derived from the in-house and GSE100197 groups (Figure 2d). The first two principal components accounted for 29.77% (PC1) and 14.08% (PC2) of the total variance. As anticipated, the PCA revealed a clear separation between the FGR and control samples, primarily along the PC1 axis. This separation confirms that the selected DMPs captured a substantial portion of the underlying epigenetic variation associated with FGR. Importantly, this pattern was consistent across both the in-house and public (GSE100197) datasets, supporting the robustness and reproducibility of these DMPs in distinguishing FGR from control cases.

### 2.3. DMPs Common Between the Groups Are Enriched in Intergenic Regions

To approximate the importance of the identified subset of common DMPs, we first analyzed the enrichment of these DMPs in specific regions of the genome, using the LOLA framework. As shown in Supplementary Table S2, the analyzed DMPs were significantly enriched in intergenic regions of the genome (FDR ≤ 0.05, OR > 2). However, we did not observe enrichment in any specific chromosome or CpG island region (FDR > 0.05). Moreover, we did not identify any enriched Gene Ontology (GO) biological process or molecular function, using GREAT and FUMA GWAS (see Section 4 for details).

### 2.4. The Genes Harboring Methylation Changes Within Promoter Regions Are Directly Related to Clinical Features of FGR

**Figure 3:**
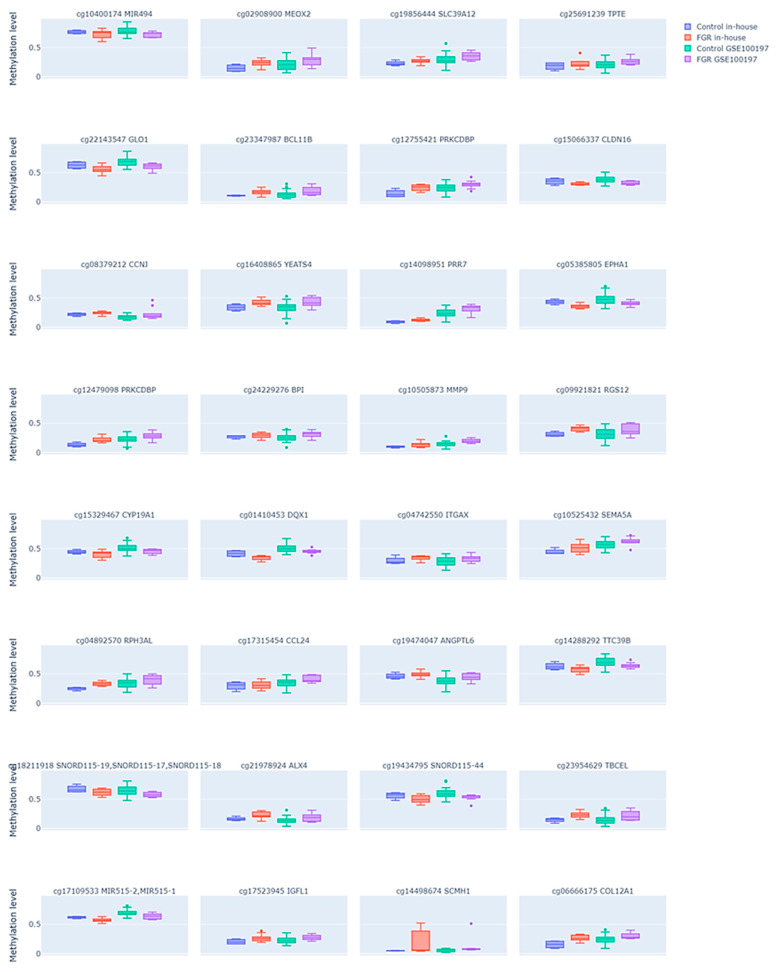
Differential DNA methylation levels in the selected subset of CpG sites in the fetal growth restriction (FGR) and control placental samples across two datasets. Each plot compares the methylation patterns between the FGR and control groups in both an in-house dataset and the public GSE100197 dataset. Each subplot is labeled with the corresponding CpG site ID and its annotated gene. Dots represent individual samples.

Since methylation changes that influence gene expression changes are usually assumed to be localized in the promoter regions of the gene, we further aimed to select only those DMPs that were placed in the transcription start site (TSS) or 5′UTR regions of a genome or directly annotated as promoters. We identified 32 such DMPs (see Supplementary Table S3), among which 22 were hyper- and 10 hypomethylated in the FGR samples compared to the controls. The comparison of these DMP methylation levels between the FGR samples and controls in both study groups is shown in Figure 3. Similarly to all common DMPs, the enrichment analysis of the promoter-associated DMPs using the LOLA framework did not reveal significant enrichment in specific chromosomes or CpG island-related regions (FDR > 0.05), as shown in Supplementary Table S4.

While the identified DMPs mapped to 31 unique genes (with two DMPs located within the *PRKCDBP* locus), we performed a functional annotation of these genes using the GeneCards platform, with detailed summaries provided in Supplementary Table S5. Most of the analyzed genes were functionally associated with cell signaling and angiogenesis (e.g., *EPHA1*, *ANGPTL6*, and *SEMA5A*), immune regulation (e.g., *ITGAX*, *CCL24*, *BPI*, and *RGS1*), and transcriptional control (e.g., *BCL11B*, *ALX4*, and *SCMH1*), processes commonly implicated in FGR. In addition, we assessed whether any of these genes have been previously reported in the context of FGR, DNA methylation, or both, based on a systematic PubMed literature search using MeSH Terms (“Gene Name” AND “Methylation”; “Gene Name” AND “FGR”; “Gene Name” AND “Methylation” AND “FGR”) as shown in Supplementary Table S5. Among the analyzed genes, 23 showed a link to DNA methylation, 7 (*MEOX2*, *BPI*, *MMP9*, *CYP19A1*, *ITGAX*, *CCL24*, and *TBCEL*) showed a link to FGR, and only 1 gene, *CYP19A1*, showed a link to both DNA methylation and FGR.

**Figure 4:**
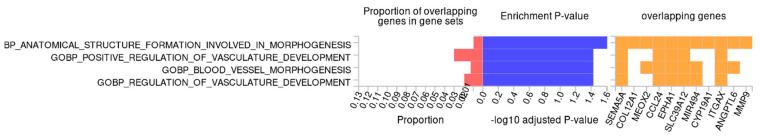
Gene ontology enrichment analysis of differentially methylated genes using Functional Mapping and Annotation of Genome-Wide Association Studies (FUMA GWAS). Enriched Gene Ontology (GO) terms are related to morphogenesis and vascular development. Bars show the proportion of overlapping genes, significance (−log10 adjusted *p*-value), and the key overlapping genes.

Finally, to approximate the biological function of the identified methylation changes, we performed functional enrichment analysis. Using GREAT, we did not observe any significantly enriched biological processes or molecular functions. However, when using FUMA GWAS, we observed that the identified methylation changes may potentially affect the genes which are involved in biological processes directly associated with the pathogenesis of FGR, such as structure formation related to developmental and vascular processes, including “anatomical structure formation involved in morphogenesis”, “positive regulation of vasculature development”, “blood vessel morphogenesis”, and “regulation of vasculature development” (Figure 4). The genes involved across all enriched biological processes were *SEMA5A*, *CCL24*, *EPHA1*, *SLC39A12*, and *ITGAX*, which can be functionally linked to inflammatory regulation, as well as angiogenic and morphogenetic signaling.

## 3. Discussion

Although numerous studies have investigated the association between fetal growth restriction and alterations in DNA methylation, their findings remain heterogeneous and, in many cases, contradictory. Differences in study design, sample source, analytical platforms, and cohort characteristics likely contributed to the variability observed across published results. To address these challenges and enhance reproducibility, we performed a comparative analysis using our in-house dataset alongside the publicly available dataset (GSE100197), which provided access to raw data and shared a compatible study design that enabled meaningful cross-cohort comparison. The materials and methods used in both studies were largely comparable. In our study, chorionic villi samples were collected from the fetal side of the placenta, consistent with the approach described for the publicly available dataset GSE100197. This methodological alignment minimized the variability introduced by differences in the region of placental sampling and ensured that both cohorts represented comparable functional compartments of the placenta. In addition, although our study utilized the Infinium Methylation EPIC, whereas the GSE100197 dataset was generated using the 450K array, both datasets were re-processed from raw IDAT files using an identical normalization and quality-control pipeline; the only analytical difference was the inclusion of GA adjustment in our cohort, which was necessary due to the early-onset nature of the FGR cases. While differences in array design limited the number of CpG sites available for direct comparison, restricting analyses to probes shared across both platforms increased the reliability and technical robustness of the cross-cohort results.

The analysis revealed a number of common differentially methylated CpG sites, with a substantial number localized within promoter regions of genes involved in angiogenic and morphogenetic signaling (e.g., *EPHA1*, *ANGPTL6*, and *SEMA5A*), immune and inflammatory regulation (*ITGAX*, *CCL24*, *BPI*, and *RGS1*), and transcriptional control (*BCL11B*, *ALX4*, and *SCMH1*). These biological processes are essential for establishing and maintaining effective maternal–fetal exchange, and their disruption is a recognized hallmark of placental pathology in FGR [30,31]. Aberrant villous branching, insufficient trophoblast invasion, and inadequate vascular remodeling lead to hypoxic stress and impaired nutrient transport, reflecting a failure of adaptive placental mechanisms [32]. Among the well-established genes associated with FGR and angiogenic imbalance are *MMP9*, *EPHA1*, *ITGAX*, *ANGPTL6*, and *CYP19A1*. *MMP9* encodes a zinc-dependent matrix metalloproteinase that is crucial for extracellular matrix degradation and trophoblast invasion, and reduced MMP9 activity has been linked to shallow placentation and impaired spiral artery remodeling [33]. *EPHA1* and *ITGAX* contribute to endothelial signaling and cell adhesion during villous morphogenesis, whereas *ANGPTL6* acts as a proangiogenic mediator, promoting endothelial proliferation and migration [34,35].

Among the identified genes, *CYP19A1* is the only one for which methylation has been previously shown to be associated with FGR [29]. *CYP19A1* encodes placental aromatase, which plays a central role in the biosynthesis of estrogens and, therefore, is essential for uteroplacental blood flow, vascular adaptation, and trophoblast differentiation. Notably, altered aromatase activity has been reported in pregnancies complicated by preeclampsia [36] and FGR [37]. An important aspect of *CYP19A1* biology is the presence of sex-specific expression patterns, with female placentas exhibiting higher *CYP19A1* expression than male placentas [38], which may partly contribute to the greater resilience of female fetuses to adverse intrauterine conditions. Although some studies have suggested region-specific hypermethylation of *CYP19A1* in particular FGR subtypes (especially among male fetuses), our analysis revealed a consistent decrease in *CYP19A1* promoter methylation across two independent FGR cohorts, including an in-house cohort composed exclusively of early-onset FGR. This reproducible hypomethylation suggests a compensatory epigenetic mechanism, potentially aimed at increasing aromatase expression to support estrogen-dependent vascular function in the context of placental compromise. Although the FGR groups displayed a slightly higher proportion of female fetuses than controls, the robustness of the hypomethylation signal across both datasets indicates that this effect is unlikely to be explained solely by sex distribution and instead represents a core epigenetic feature of early-onset FGR.

Interestingly, we also identified several genes not previously reported in the context of fetal growth restriction, but potentially relevant to placental development and function, such as *SLC39A12*, *BCL11B*, *YEATS4*, and *SEMA5A*, as well as small RNAs, such as *MIR494*, the *MIR515* family, and the *SNORD115* family. Among these, *BCL11B* and *YEATS4* act as transcriptional regulators involved in chromatin remodeling and may influence trophoblast differentiation [39,40]; *MEOX2*, a mesodermal transcription factor, plays a crucial role in endothelial differentiation and vascular maturation [41], and *SLC39A12*, encoding a zinc transporter, suggests a potential link between metal ion homeostasis, oxidative stress, and placental insufficiency [42]. Furthermore, *SEMA5A* and *CCL24* are involved in immune signaling and vascular guidance, pathways increasingly recognized as critical in maintaining immune tolerance and vascular integrity at the maternal–fetal interface [43,44].

Of note, the identification of microRNAs, such as MIR494 and members of the MIR515 family, together with *SNORD115* transcripts, suggests that post-transcriptional gene regulation may interplay with epigenetic regulation as an additional layer of FGR-related placental pathology [45,46]. These small RNAs may modulate angiogenesis, trophoblast invasion, and endothelial function by targeting key signaling pathways, including the VEGF pathway, which regulates vascular permeability and new vessel formation; the Notch pathway, which governs cell fate decisions and trophoblast differentiation; and the HIF (hypoxia-inducible factor) pathway, which mediates cellular adaptation to low oxygen tension and plays a central role in placental vascular remodeling.

Several limitations of the present study should be acknowledged. Although two independent cohorts were included, the overall sample size remains relatively modest and partially imbalanced, which may reduce statistical power and limit the detection of more subtle methylation differences. In addition, despite reprocessing both datasets from raw IDAT files using a uniform normalization and quality-control pipeline, residual batch effects related to differences in array platforms (EPIC versus 450K), sample processing, and cohort-specific characteristics cannot be fully excluded. Furthermore, the scope of the present study was restricted to DNA methylation profiling, and no functional validation experiments were performed to directly assess the biological consequences of the identified epigenetic alterations. Finally, although key clinical variables were considered where available, incomplete maternal and environmental data across cohorts precluded comprehensive adjustment for all potential confounders.

## 4. Materials and Methods

### 4.1. Study Groups Characteristics

In our study, we analyzed placental tissue from 16 pregnancies of patients who delivered at the Clinical Department of Obstetrics and Gynecology, Pomeranian Medical University in Szczecin, Poland, between February 2020 and January 2022. All samples were collected according to the protocol approved by the institutional review boards, with written consent obtained from each patient (KB-0012/122/12)

The gestation at delivery ranged between 26 and 39 weeks of gestation. In total, 11 pregnancies were affected by the early-onset FGR, and 5 were unaffected and thus considered the control group. FGR was identified in utero based on Delphi consensus ultrasound criteria [1] and confirmed at birth. Exclusion criteria included fetal chromosomal abnormalities, intrauterine infections, multifetal pregnancies, maternal age < 18 years, psychiatric disorders, or lack of consent. Clinical characteristics of the patients are presented in Table 1.

To increase the statistical power of the study, as well as identify methylation changes common between different study groups, we searched for other studies showing an association between DNA methylation changes and FGR. Among seven studies identified [18,19,20,21,22,23,24], raw data were available only for one cohort [24]. This dataset was analyzed alongside our cohort to compare methylation changes characteristic of FGR.

Publicly available DNA methylation data for this cohort were obtained from the Gene Expression Omnibus (GEO) under accession number GSE100197 [24]. This dataset originates from a study investigating placental DNA methylation alterations associated with preeclampsia and intrauterine growth restriction (IUGR) using the HumanMethylation450 BeadChip (Illumina, San Diego, CA, USA). Placental chorionic villi samples were collected from multiple placental sites and pooled to obtain a representative methylation profile. The full GSE100197 series includes placentas from normotensive controls as well as pregnancies complicated by early-onset preeclampsia (EOPE), late-onset preeclampsia (LOPE), and normotensive IUGR. In the present study, analyses were restricted to IUGR cases (hereafter referred to as FGR, in accordance with current terminology) and normotensive controls (11 FGR and 43 controls), in order to maintain clinical homogeneity and enable direct comparison with the in-house cohort.

### 4.2. Sample Characteristics

Chorionic villi samples were obtained from the fetal side of the placenta. The samples from four sites (0.05 g each) of the placenta were pooled, frozen within 2 h of sampling at −80 °C, and stored until DNA extraction.

### 4.3. DNA Extraction and Initial Preparation

Genomic DNA was extracted using the standard salting out method as described previously [47]. The quantity of obtained DNA was assessed using Qubit™ dsDNA BR Assay and Qubit^®^ 2.0 Fluorometer (Invitrogen, Waltham, MA, USA). Bisulfite conversion of DNA (500 ng) was carried out using EZ DNA Methylation-Gold Kit (Zymo Research, Irvine, CA, USA), according to the manufacturer’s protocol.

### 4.4. Genome-Wide Methylation Analysis

Methylation level assessment was performed using commercial Infinium Methylation EPIC v1.0 (Illumina, San Diego, CA, USA) microarrays. Raw IDAT files were imported into R (version 4.3.1), and initial quality control was conducted using the MethylAid package [48], which identifies low-quality samples and outliers based on signal intensity distributions and control probe metrics across arrays. Based on this quality-control step, three samples from the GSE100197 dataset were excluded from further analysis.

Subsequently, raw methylation data were processed using the standard ChAMP pipeline [49]. Probe filtering steps implemented within ChAMP were applied to minimize technical bias and included the removal of probes with detection *p*-values > 0.01, probes containing single-nucleotide polymorphisms, non-CpG probes, cross-reactive probes, as well as probes located on sex chromosomes (X and Y).

After initial preprocessing, our dataset contained methylation data (beta-values) for a total number of 745,656 probes for in-house generated data and 418,756 probes for the GSE100197 dataset. All downstream analyses were restricted to the probes common between these datasets (n = 377,995).

### 4.5. Estimation of Cell-Types Proportions in Analyzed Tissues

As each cell type has a different methylome, to identify causal methylation pattern alterations in individual cell types, the correction for the proportion of different cells in the individual sample should be performed [50,51,52,53]. To infer the proportions of cell types present in the analyzed samples, we used the robust partial correlation (RPC) algorithm implemented in the EpiDISH package, using R version 4.3.1 [54] and the “plCellCpGsThird” reference methylation profiles from the planet package [55]. Estimated cell proportions were used to detect samples with abnormal composition, using the local outlier factor algorithm implemented in the scikit-learn library.

### 4.6. Identification of Methylation Changes Potentially Attributed to FGR

In order to identify methylation changes between cases and controls, we used univariate or multivariate linear regression models, depending on data availability and cohort characteristics. For the publicly available GSE100197 dataset, univariate models were used, as key clinical covariates, such as gestational age and fetal sex, were not significantly different between cases and controls and were not identified as potential confounders in this cohort. In contrast, multivariate models were applied to the in-house generated dataset to adjust for GA, which was significantly different between cases and controls (*p*-value ≤ 0.05, Kruskal–Wallis test). Finally, we defined differentially methylated positions (DMPs) as a position with an estimated absolute methylation level difference ≥ 0.05 and *p*-value (*t*-test) ≤ 0.05.

### 4.7. Functional Analysis

To approximate the biological processes potentially affected by the identified changes in methylation, we used the Genomic Regions Enrichment of Annotations Tool (GREAT) version 4.0.4 tool and Functional Mapping and Annotation of Genome-Wide Association Studies (FUMA GWAS [56], accessed in 2024). The annotation to genomic regions was performed using the Locus Overlap Analysis (LOLA) framework [57] with a custom database created using the EPIC B5 manifest. As a background for enrichment analysis, we used a set of all analyzed genomic intervals (n = 377,995).

### 4.8. Statistical Analysis

Statistical analyses were performed in R 4.3.1 and Python 3 environments. Regression models were estimated using the ordinary least squares estimator. Associations between the binary phenotype and potential confounders were analyzed using Kruskal–Wallis or Fisher’s exact tests for continuous and categorical variables, respectively, with the level of significance as 0.05 (alpha). All figures were generated using Plotly.js (v2.27.0).

## 5. Conclusions

In conclusion, our study highlights the value of cross-cohort analyses in identifying conserved epigenetic signatures of FGR. By focusing on reproducible epigenetic alterations across independent datasets, our findings strengthen the evidence for the involvement of DNA methylation in placental dysfunction associated with FGR. Future research should prioritize multicenter collaborations, standardized methodologies, and larger, well-characterized cohorts to validate and extend these observations. Such efforts are essential for advancing our understanding of the epigenetic mechanisms underlying placental dysfunction and fetal growth restriction.

## Figures and Tables

**Table 1:** Clinical characteristics of the patients.

	FGR	Controls
Age, years	32.5 ± 3.2	32.0 ± 5.7
Height, cm	166.5 ± 6.3	165.2 ± 8.9
Weight, kg	68.6 ± 13.9	57.4 ± 1.6
BMI, kg/m^2^	24.8 ± 4.7	21.2 ± 2.2
Gravidity	3 (2–4)	3 (1–3)
Parity	2 (1–3)	1 (1–1)
GA, weeks	30.9 ± 3.2	38.5 ± 0.6
Hypothyroidism	3 (27%)	3 (60%)
Preeclampsia	5 (45%)	0 (0%)
Mode of delivery		
Vaginal	0 (0%)	1 (20%)
Cesarean	11 (100%)	4 (80%)
Neonatal birth weight, g	1211 ± 526	3410 ± 232
Apgar < 8 at 5 min	3 (27%)	0 (0%)

Values are given as mean ± SD, median (range), or n (%). FGR—fetal growth restriction, BMI—body mass index, GA—gestational age.

## Data Availability

Methylation profiling data are deposited in the NCBI Gene Expression Omnibus (GEO; https://www.ncbi.nlm.nih.gov/geo/; accessed on 23 December 2025) database under accession number GSE314658, and will be released upon publication.

## Supplementary Materials

The following supporting information can be downloaded at: https://www.mdpi.com/article/10.3390/ijms27031448/s1.

## Abbreviations

The following abbreviations are used in this manuscript:

BR	Broad range
CpG	Cytosine–phosphate–Guanine
DMPs	Differentially methylated positions
DNA	Deoxyribonucleic Acid
EPIC	Infinium Methylation EPIC BeadChip
FDR	False discovery rate
FGR	Fetal growth restriction
FUMA GWAS	Functional Mapping and Annotation of Genome-Wide Association Studies
GA	Gestational age
GO	Gene Ontology
GREAT	Genomic Regions Enrichment of Annotations Tool
GSE	Gene Expression Omnibus Series
IDAT	Illumina data file format
LOLA	Locus Overlap Analysis
nRBC	Nucleated red blood cell
OR	Odds ratio
PCA	Principal component analysis
RPC	Robust partial correlation
TSS	Transcription start site
