# Supplementary material for: Integrative Analysis of Placental Methylomes Identifies Epigenetically Regulated Genes Implicated in Fetal Growth Restriction
Source: Int J Mol Sci. 2026 Jan 31;27(3):1448. doi: 10.3390/ijms27031448 (PMC12897614; doi:10.3390/ijms27031448)
Supplement: Supplementary file 1 [file ijms-27-01448-s001.zip › ijms-4079256-supplementary Figure S1.pdf]

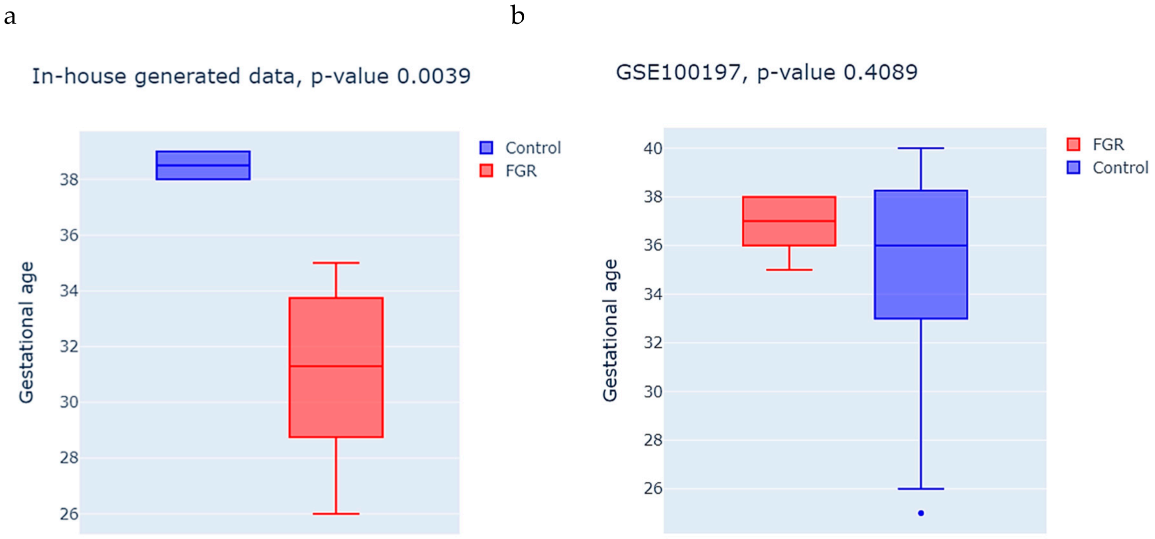

Supplementary Figure S1. Comparison of gestational age between FGR and controls in both datasets. (a) In-house data: a significant difference in gestational is observed between groups, with FGR samples showing a lower GA compared to controls. (b) Public data (GSE100107): no significant difference in gestational age is detected between the groups (p = 0.4089), with considerable overlap in distributions.
